# Impacts of Wildflower Interventions on Beneficial Insects in Fruit Crops: A Review

**DOI:** 10.3390/insects13030304

**Published:** 2022-03-18

**Authors:** Michelle T. Fountain

**Affiliations:** NIAB EMR, East Malling, Kent ME19 6BJ, UK; michelle.fountain@niab.com

**Keywords:** agroecology, agri-environment schemes, habitat, landscape, production, sowing

## Abstract

**Simple Summary:**

Declines in insects, including pollinators, are leading to public pressure for governments and landowners to intervene. Although integrated pest management, used to preserve the natural enemies of pests, has been in place for decades, promoting the preservation of pollinators is more recent, especially on farmed land. To enable fruit production, which is less environmentally damaging, natural enemies and pollinators are key. In addition, a reduction in available pesticides is forcing fruit growers to reconsider their management practices. One intervention that is increasingly being used is the provision of floral resources as wildflower areas or cover crops to encourage natural enemies and pollinators. This review brings together the literature on the benefits and costs of provisioning flora in the vicinity of fruit crops. It highlights that most impacts of floral resource provision in the vicinity of fruit crops are beneficial or benign.

**Abstract:**

Integrated pest management (IPM) has been practiced by the fruit industry for at least 30 years. Naturally occurring beneficial insects have been encouraged to thrive alongside introduced predatory insects. However, Conservation Biological Control (CBC) and augmented biocontrol through the release of large numbers of natural enemies is normally only widely adopted when a pest has become resistant to available conventional pesticides and control has begun to break down. In addition, the incorporation of wild pollinator management, essential to fruit production, has, in the past, not been a priority but is now increasingly recognized through integrated pest and pollinator management (IPPM). This review focuses on the impacts on pest regulation and pollination services in fruit crops through the delivery of natural enemies and pollinating insects by provisioning areas of fruiting crops with floral resources. Most of the studies in this review highlighted beneficial or benign impacts of floral resource prevision to fruit crops. However, placement in the landscape and spill-over of beneficial arthropods into the crop can be influential and limiting. This review also highlights the need for longer-term ecological studies to understand the impacts of changing arthropod communities over time and the opportunity to tailor wildflower mixes to specific crops for increased pest control and pollination benefits, ultimately impacting fruit growers bottom-line with less reliance on pesticides.

## 1. Introduction

**Figure 1:**
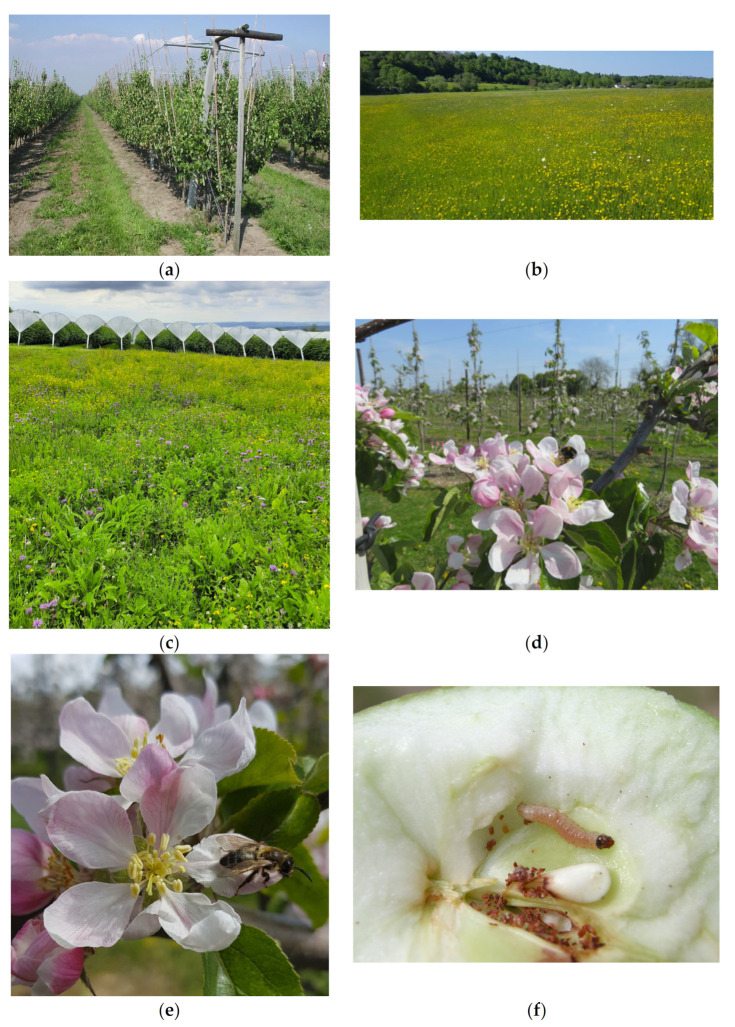
(**a**) Intensively grown pear orchard with low floral diversity (credit NIAB EMR), (**b**) semi-natural wildflower meadow (credit Konstantinos Tsiolis), (**c**) wildflower planting on margin of polytunnel grown raspberries (credit Celine Silva), (**d**) bumblebee foraging on an apple blossom, (**e**) andrenid bee on an apple flower, an important pollinator of the apple (credit Konstantinos Tsiolis), and (**f**) codling moth larvae in an apple (credit NIAB EMR).

Post WWII there was a drive to intensify agriculture (Figure 1a) with a transition from traditionally diverse agro-ecosystems to industrial modes of agriculture with simplified and chemically-dependent agricultural management, which increased yields but at a cost to the environment, including beneficial insects and the ecosystem services they provide [1,2]. Natural England [3] estimated by 1984, in lowland England and Wales, that semi-natural grassland had declined by 97% over the previous 50 years and only 7500 ha remained by 2010. Losses continued during the 1980s and 1990s at a rate of 2–10% per annum in parts of England.

Floral resources are generally implemented to counteract the lack of food and nesting resources to support natural enemies, pollinators, and biodiversity in the local landscape. Currently, integrated pest management (IPM), which aims to minimize and integrate the use of pesticides, is part of the legislation with National Actions Plans commissioned (e.g., Defra [4]). IPM requires additional effort in monitoring, the use of prediction tools, biological controls, and expert knowledge. In more recent years, the withdrawal of pesticide approvals and the development of pesticide resistance, combined with increases in exotic pests and diseases, has incentivized farmers and growers to engage and implement IPM practices. 

IPM methods aimed at pest control might also benefit pollinating insects—key contributors to fruit growing—and improve yield and quality [5,6,7]. Indeed, IPM practices can increase crop yields through the preservation of pollinating insects [8]. Recently, Egan et al. [9] proposed the introduction of a systematic framework for integrated pest and pollinator management (IPPM). They highlighted that pest and pollinator management currently remain largely uncoordinated, offering an opportunity to boost critical pollinating insects in flowering crops and the wider landscape.

Non-pesticide strategies aimed at pest control have focused on classical biological control [10]. Biological control agents (BCA), releases of organisms used to control pests species, are not normally widely adopted until a pest has become resistant to conventional pesticides and control begins to break down (e.g., pear sucker, western flower thrips). Michaud [10] argues that BCA approaches do not constitute an ecologically sustainable solution because continued inputs are required. 

More recently, there is a move towards controlling pests through Conservation Biological Control (CBC, [11]). Before embarking on designing new habitats or modifying existing habitats to support the natural enemies and pollinators required, there is a need to understand the biology, ecology, and interactions at a habitat scale [7,12,13]. Furthermore, to make these approaches economical, it is possible to apply more targeted tactics [14], but evidence of the success needs long-term (years) implementation and close monitoring in fully replicated experiments, which requires investment [7].

Intensive land use can reduce functional species richness, but the actual species richness of generalist insect groups may be unaffected [15]. In addition, local habitat quality may only impact specialist groups and not support the functional groups required in the crop [15]. However, maintaining biodiversity can improve pest regulation and improved ecosystem resilience for future environmental changes [16,17], even if it does not always result in improved ecosystem function [18]. For example, wild pollinators are frequently more effective fruit pollinators than honeybees, adding support for the need to preserve a diverse set of wild pollinators in agroecosystems [19]. 

The estimated total area of fruit (including apples, pears, plums, and soft fruit) grown in the UK in 2010 was 34,324 ha compared to 33,639 in 2020 (provisional data Defra [20]) with 559.3 and 657.0 thousand tons produced on that land, respectively. This represents an increase in production of 97.7 thousand tons of fruit on slightly less land with almost double the value of GBP 580 million to GBP 1045 million for the UK economy in just 10 years. These modern perennial fruit crops are planted at high densities in rows, often with sown alleyways of a grass sward, typically *Lolium perenne*, *Festuca* spp., and *Poa pratensis* [21], or they are unsown to allow for natural development of the resident grass and forb community (Figure 1a).

More recently, fruit growers have begun to sow areas of wildflowers, which reportedly offer environmental and ecosystem service (goods and services that humans gain from the natural world) benefits [22,23]. A habitat that promotes beneficial insects (e.g., natural enemies and pollinators) could provide natural pest regulation and pollination [6,24,25,26,27] and also improve biodiversity [24], soil protection, water quality [26,28,29], and weed suppression [28,29,30,31,32]. Habitat improvements also enhance rural aesthetics, giving additional secondary benefits [26], which are not always considered in economic assessments. 

Cover crops generally refer to plants grown to protect and enrich the soil but might also be implemented to avoid or divert pests, alter host-plant nutrition, reduce dust and drought stress, change the microclimate, and increase natural enemy efficiency [33,34]. They can also provide a food resource for pollinators and tend to be annual or biannual agricultural plants. In contrast, perennial wildflower sowings are more complex plant assemblages, often referred to in the literature as wildflower strips, sown on the margin or within the crop (alleyways) [34] with the aim to increase the abundance and diversity of beneficial arthropods [35]. Semi-natural habitats are also included in this review, as they may be flower rich, although they tend to receive minimal intervention [36] (Figure 1b).

Currently, agri-environment schemes that encourage and subsidizes habitat manipulations do not measure successful implementation, or the benefits provided [37,38]. Although, in the UK at least, this may change with the introduction of environmental land management schemes (ELMs) [39]. In an analysis of twenty studies, Kleijn and Sutherland [37] found that 54% of species in agri-environment schemes increased and 6% decreased in species richness or abundance compared with controls. 

Most floral interventions require investment, need specialist knowledge and equipment to install (Figure 1c), and, once established, require ongoing monitoring and management to maximize benefits [40], including mowing, scarifying, or tillage to influence the floristic composition and longevity [41]. 

The aim of this review was to summarize publications on the impact of floral resources on fruit crops. The search terms ‘pollinators and fruit’, ‘natural enemies and fruit’, and then combinations with ‘wildflowers’ ‘flora’ and ‘pest’ were entered into Google Scholar. From these published articles, previous manuscripts were sourced from reference lists, and papers were tabulated into categories. Only rarely were natural enemies and pollinators considered in the same publication, even though a floral resource might benefit both within the crop [26,42].

This review focuses on the benefits that additional floral resources, in the vicinity of fruit crops, provide to pest regulation and pollination services through the provision of natural enemies (predators and parasitoids) and pollinating insects. This review summarizes: (1) The impact of farm and landscape scale floral components, and (2) the impacts of floral resources on fruit damage and production. It then poses the questions (3) does the length of time a floral resource is in place impact benefits? (4) what is the impact of wildflower intervention (vegetation) structure and composition? (5) what is the impact of floral resource size? and (6) do distance from the crop and edge impact the effectiveness of wildflowers? The review goes on to summarize (7) the benefits of floral resources to natural enemies and pollinators, and detrimental impacts on crop production, before reviewing (8) The choice of floral resources and (9) The establishment and management of floral resource, finally drawing conclusions and recommendations for future research.

## 2. Impact of Farm and Landscape Scale Floral Components

Consideration should be given to the implementation of floral resources as part of the wider landscape. Higher quality and/or areas of sown flower-rich habitats within a farm improve the abundance of bumblebees and solitary bees [43]. In a study on nine farms, with flower strips along a gradient of landscape heterogeneity and farming intensity, solitary bees declined with increasing distance from flower strips, but only in more complex landscapes [44]. Bumblebees, but not solitary bees, increased in abundance in field borders outside flower strips in floristically-enhanced landscapes compared with landscapes that did not have additional flower strips [44], most likely because bumblebees can forage for greater distances than solitary bees [45]. However, wild bee abundance decreased by 48%, species richness by 20%, and strawberry fruit counts by 18% across farms provisioned with honeybee hives, regardless of wildflower strip presence [46]. Bumblebees (Figure 1d) foraged at shorter distances where local landscape had a high cover and low fragmentation of seminatural vegetation, including managed agri-environmental field margins, although the effect was bumblebee species dependent [45]. 

## 3. Impacts of Floral Resources on Fruit Damage and Production

Fewer than 15 studies in this review followed implementation of flower-rich interventions through to economic or production impacts on neighboring fruit crops, and only some of these outcomes related to the floral intervention directly. 

### 3.1. Natural Enemies

Pest regulation services in orchards can be improved with wildflower alleyways. In apples, where no insecticides were applied for five years, plots with wildflower alleyways had 9.2% damaged fruits compared to 32.5% damaged fruits in (no manipulation) controls. This was primarily due to reduced tarnished plant bugs and summer Lepidoptera damage, and it was because wildflowers attracted and retained beneficial arthropods that effectively managed several apple pests [47]. However, in UK commercial apple orchards, McKerchar et al. [48] showed that the presence of wildflower strips in alleyways did not contribute to the delivery of natural pest regulation, even though hoverfly diversity and species richness were greater in orchards with wildflower strips. This was attributed to cumulative pesticide toxicity negatively affecting natural enemy populations, especially earwigs [48]. 

Although Markó et al. [49] showed no impact on fruit injury by insect pests, including codling, *Cydia pomonella* (Figure 1f), and tortrix moths in apple orchards with wildflower alleyways, Fountain et al., (unpublished) recorded reduced damage by these pests in apple orchards with wildflower alleyways in combination with earwig refuges and semiochemical hoverfly attractants in the trees. Altieri and Schmidt [29] also recorded fewer codling moths in orchards with floral alleyway sowings (36.1% infested apples compared to 45.0% in the control plots). 

Fruit damage was reduced in organic apple orchards with floral alleyways attributed to slower *D. plantaginea* population increase and the promotion of aphidophagous and generalist predators [50]. Species richness of beneficial arthropods in organic apple orchards, normally associated with a higher abundance of flowering plants, was not correlated with fruit production, suggesting that diversity could be increased without large yield losses [51]. In addition, the most productive organic orchards exceeded the mean yields of IPM orchards, but fruit damage at harvest was higher in organic orchards, creating an indirect negative effect. 

### 3.2. Pollinators

Although a meta-data analysis (synthesis of 23 studies—representing 16 crops on five continents) of the relationship between pollination services and distance from natural or seminatural habitats provided evidence of decreasing crop visits and pollinator richness with distance from a floral rich natural habitat, there was less evidence of a decline in fruit and seed set (variables that directly affect yields) [36]. However, there was significantly higher fruit production in mango crops near to native wildflowers. Yield increased by 15 kg of commercially saleable mango per tree attributed to a higher diversity and abundance of mango flower insect visitors [52]. 

Cherry blossoms visited by insects produced 30.2% more marketable fruit compared to only 1.4% if insects were excluded (using excluding mesh bags). However, supplementary hand-pollinated blossoms achieved 51.7% marketable fruit, indicating a significant pollination deficit in the studied orchards [40]. In another study, where pollinator species richness and wild pollinator abundance had a positive influence on a fruit set of sweet cherry, the link to floral interventions was less clear [53]. Holzschuh et al. [54] concluded that an increase from 20% to 50% of high-diversity bee habitats in the landscape enhanced the fruit set in cherry orchards by 150% because wild bee visitation to cherry blossoms increased with the proportion of high-diversity bee habitats in the surrounding landscape. The value of pollinating insects to sweet cherry in the UK is estimated at GBP 11.3 million (£14,731.8 ha^−1^), while this could be increased to GBP 25,608 ha^−1^ if pollination management was improved [40]. 

Avocado yield increased by 40–60% in fields next to native flower habitats, which was attributed to increases in the number of flies that were responsible for pollination [55].

Wild bees are particularly important to apple production and can negate crop losses when honeybees are distracted by other co-flowering crops such as OSR [56]. A fruit set of cider apples was positively related to wild insect flower visitor richness and andrenid bees (ground nesting solitary bees) but not flower strips, even though visit rate to apple blossoms of wild bees and Diptera increased to 40% in orchards with wildflower alleyways [57]. Likewise, dessert apples had a higher fruit set where the species richness of wild bees (Figure 1e) was higher, regardless of the presence of honeybees [43]. The fruit set responded positively to a higher abundance and richness of wild bees, whereas the seed set depended on the abundance of wild pollinators in cider apple orchards [58]. Insect flower visitation rates were higher in organic orchards compared to IPM orchards, resulting in a positive impact of organic management on apple production [51].

Fruit set, weight [59], and mature seeds (Garratt et al., submitted) can be positively impacted by insect visits. However, a more recent study found fewer seeds in apples with enhanced floral landscapes and no consistent improvement in fruit quality or yield [60].

Strawberry fruit yields were lower when honeybees were installed on a farm where wild bee abundance and diversity also decreased [46]. Strawberries grown in landscapes with well-connected semi-natural habitats increased in commercial value from EUR 9.27 per 1000 strawberries, compared to plants grown with grassy margins to EUR 14.95, through increased yield and quality, most likely facilitated through easier movement of pollinators through the landscape to the crop [61]. 

Fruit quality was significantly greater in blueberry fields adjacent to wildflower plantings, three and four years after establishment, leading to higher crop yields. In addition, the increased associated revenue exceeded the cost of wildflower establishment and maintenance [62]. 

Wildflower margins can be a part of increasing landscape complexity. Mateos-Fierro et al. [40] concluded that wildflower plantings in orchard alleyways are an effective approach to enhance ecosystem services delivered by natural enemies and pollinators in orchards that could reduce pesticide inputs and increase yields, subsequently increasing profits to growers. However, fruit growers need to couple floral interventions with a careful selection of pesticide applications [48].

## 4. Does the Length of Time a Floral Resource Is in Place Impact Benefits?

Of over 130 papers reviewed, fewer than 30 had long-term, three years or more, observations. Given the time for perennial floral margins to establish and arthropods to colonize and diversify, studying over a longer period is key to interpreting the impact that floral interventions have on cropping systems. Studies that are shorter than four years may miss long-term benefits. In arable landscapes, habitat creation, including wildflower resources, increased yields, but it took around four years for the beneficial effects on crop yield to be realized, with effects becoming more pronounced over six years [63].

The economic benefits of floral margins in highbush blueberries were only seen in years 3–4 after establishment, where wild bee and hoverfly abundance increased annually in fields adjacent to wildflower plantings [62]. In Quebec apple orchards, significantly higher percentages (98%) of undamaged fruits were only recorded five years after sowing, and several seasons were required to build up populations of beneficial insects to achieve effective pest control [47]. Populations of predators (mainly spiders) and predator–prey ratios were also higher in six- compared to one-year-old floral strips [30].

In unsprayed apple orchards, after five years, there was 9.2% fruit damaged by the tarnished plant bug and summer Lepidoptera in floral treatments (sown flower mix) compared to 32.5% damage in the control (no manipulation) orchards [47]. Several seasons were needed to build up beneficial insects, but additional flora attracted and retained beneficial insects that effectively managed several arthropod pests [47]. In another five-year study, no significant effects from the presence of flowering weeds (primarily wild carrot, parsnip, hogweed) were observed on the prey–predator or host–parasite relationship in cider apple orchards [64]. This may have been because the alleyways were not purposely sown with a tailored mix to improve the diversity of floral resources. Indeed, simply allowing the resident flora of alleyways to grow unhindered in apple orchards can result in pest problems (e.g., encouraging pernicious weeds such as dock can promote damage by dock sawfly to fruit (pers. obs.)). 

Cover crops of summer savory (Satureja hortensis), ageratum (Ageratum houstonianum), and basil (Ocimum basilicum) in pear orchards decreased Psylla chinensis, Aphis citricola, and Pseudococcus comstocki as their natural enemies (Coccinella septempunctata, Phytoseiulus persimilis, and Chrysoperla sinica) increased [65]. Within the first year of floral alleyway establishment, in newly planted apple orchards (Fountain et al., unpublished), there was reduced occurrence of spring aphids, a reduction in the codling moth, *C. pomonella*, and fewer damaged apples. This was coupled with an in hoverflies and lacewings. However, in this study, hoverfly attractants and overwintering refuges were also employed and could have played a role. 

**Figure 2:**
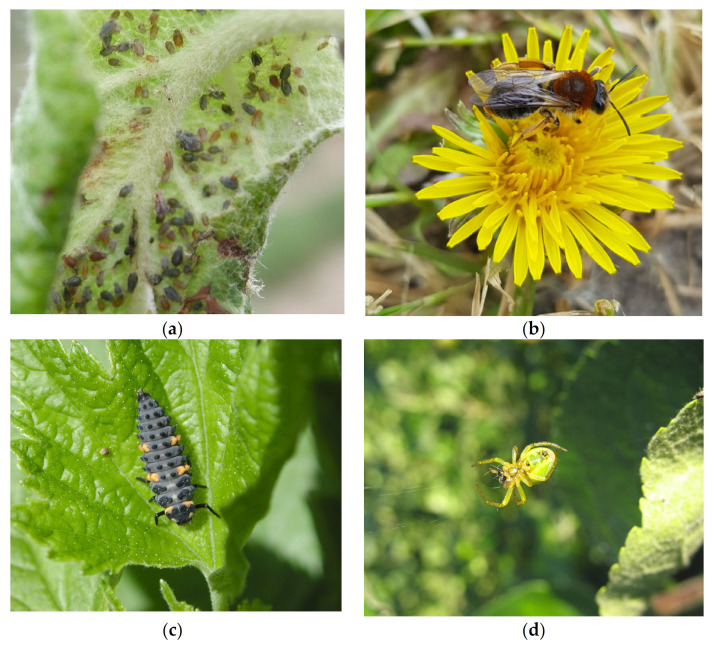

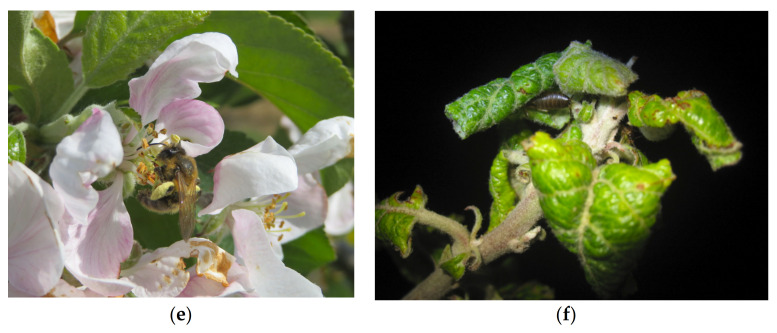
(**a**) Rosy apple aphid colony on an apple (credit NIAB EMR), (**b**) *Andrena haemorrhoa*, a ground nesting solitary bee, on a dandelion (credit Konstantinos Tsiolis), (**c**) ladybird larva (credit NIAB EMR), (**d**) foliage dwelling spider (credit NIAB EMR), (**e**) solitary bee visiting an apple flower (credit NIAB EMR), and (**f**) European earwig in an apple aphid colony at night (credit Csaba Nagy).

Natural enemies increased from 73.9% and 12.9% in alleyways and trees, respectively, in alleyways managed for wildflowers compared to regularly mown alleyways within three years of establishment in protected cherry orchards [40]. McKerchar et al. [48] demonstrated an increase in hoverfly diversity and species richness in apple orchards with wildflower alleyways, but there was no impact in the three-year study on aphid removal from bait cards and no decrease in pests (rosy apple aphid, (Figure 2a), woolly apple aphid), attributed to pesticide applications decreasing earwig numbers. In contrast to earwigs, more mobile arthropods, such as ‘ballooning’ spiders, might respond more rapidly with floral alleyways and margins significantly reducing the numbers of aphids returning to the trees in the autumn [66,67].

In a six-year study of pests and natural enemies in apple orchards, Markó et al. [49] found no additional control of pests in alleyway sowings of perennial flowering herbs, although the abundance and diversity of predatory phytoseiid mites increased with the flowering ground cover in spring and autumn. In addition, *Typhlodromus pyri* gradually displaced *Amblyseius andersoni* in the presence of flowers. Although lacewing, parasitoid wasps, and spider numbers were higher in flower-sown orchards compared to control orchards, no significant increase in pest control was observed at this time [68,69]. Markó et al. [49] also pointed out the impact of several highly active insecticides disguising any potential effects of natural biocontrol. 

Pollinators can take time to respond to floral provision because many species have only one generation a year. However, more pollinators were recorded in cherry orchards with managed floral alleyways (maintained at a 20 cm height) compared to regularly mown alleyways, leading to a 6.1% increase in the fruit set within three years [40]. The greater floral resource also led to an increase in pollinating insects in summer compared to unsown alleyways, supporting pollinators after cherry flowering. The abundance and species richness of three species of bumblebee queens increased in orchards with three-year-old enriched habitats, including wildflower margins [60]. In contrast, we saw an uplift in the number of solitary bees on apple blossoms at sites with flower plots within the first year (Garratt et al. submitted), although the extent of this uplift was not significantly different between year 1, 2, and 3 after flower establishment. In the same study, we observed species of solitary bees utilizing the sown flower margins, which were also key apple pollinators [70], suggesting that floral provision enhances the numbers of apple pollinators within the first year of flowering. 

## 5. What Is the Impact of Wildflower Intervention (Vegetation) Structure and Composition?

Pollinators and natural enemies require food (pollen, nectar, prey, etc.) for nutrition, which may include different resources depending on life stage, physiological state, time of year, etc., from a diverse range of sources. 

Sward architecture, plant diversity, and species richness are key to proving resources required by beneficial arthropods [1,71,72,73]. In observations of a gradient of grassland plant species richness (73 plots), pollinator visits increased linearly with both blossom cover and the number of flowering plant species [74]. In addition, plant species enhanced the temporal stability of flower visits [74,75], and the impact of floral resources can be increased by contrasting what is sown with what exists in the local environment [72].

Nutritional quality is key when selecting plants for wildflower strips. In a UK study of flower meadows, the nutritional status (pollen and nectar) of flowers in annual and perennial seed mixes was compared to weed species. Flowers that provided the highest rewards included *Leontodon hispidus*, *Centaurea cyanus, C. nigra*, and dandelion (*Taraxacum* spp. (Figure 2b)) for nectar and *Papaver rhoeas*, *Eschscholzia californica,* and *Malva moschata* for pollen [76]. Wildflower meadows provide resources later in the season, with pollinators relying on weed species for early forage. Early pollen and resources are especially important for solitary bees, whereas bumblebees require forage for a longer period through the season [72], and floral species richness is key to increasing bee nesting near orchards [77]. 

Food resources need to be connected to the nesting habitat (for reproduction), nesting material, structure (e.g., web-building spiders), and shelter sites (for overwintering, protection from weather, predation, etc.). For, example, the diversity of solitary wild bees is limited not only by floral resources but also by their nesting habitat [78].

## 6. What Is the Impact of Floral Resource Size?

Dicks et al. [79], tentatively suggested that a 2% flower-rich habitat and a 1 km flowering hedgerow were sufficient provision for six common pollinator species, depending on wildflower mix quality. However, Heard et al. [80] detected no impact of sown legume and grass mix patch size (including 0.25, 0.5, and 1.0 ha) on bumblebee density. Westphal et al. [81] demonstrated that bumblebee numbers were not determined by the proportion of semi-natural habitats in agricultural landscapes, but by the availability of highly rewarding mass flowering crops (i.e., oilseed rape), and they highlighted the need for landscape scale management schemes. In contrast, semi-natural habitats were key to wild bee diversity in agricultural landscapes, with floral strips offering only a partial substitute [82]. However, purposely sown flora offer a higher flower abundance than some semi-natural grasslands and are often more pollinator rich [83].

In a study on 344 fields from 33 pollinator-dependent crops in Africa, Asia, and Latin America, it was concluded that increasing floral provision for pollinators in small compared to large cropping areas had a greater impact and improved yields [84]. Indeed, wild bees were the dominant pollinators in small blueberry fields (58% of flower-visiting bees) compared to large blueberry fields (97% honeybees) [85]. 

Several small fragments of flower-rich habitats can also support more butterfly and parasitoid species than the same area composed of only one or two fragments [86]. Parasitism also improved with increased fragment area (either several small or one large area). These authors suggested that small habitat areas should be scattered to maximize diversity and minimize the risk of species loss [86]. Kremen et al. [87] suggested that a 10% upland habitat could provide 20–40% of pollination needs for watermelon and potentially benefit other fruit crops [88]. Natural enemy density, group richness, and diversity increased, and pest aphids decreased in crops (soybean) provisioned with wildflower plot sizes from 1 to 100 m^2^ (Blaauw and Isaacs, 2012).

## 7. Do Distance from the Crop and Edge Impact the Effectiveness of Wildflowers?

The provision of floral margins improves bee diversity in adjacent crops compared to crops with no marginal pollen and nectar provision [89], but the proximity of floral strips to crops and the mobility of the target arthropod has an impact on what floral interventions can deliver. Nevertheless, although edge responses are deemed predictable and consistent [90], data for consistent and reliable positive edge responses to drive pest control and pollination services have not been synthesized. Hedgerow and floral margins create a spill-over of organisms into managed cropping areas [91], but by providing floral resources within the crop, e.g., alleyways, we anticipate that the spill-over will occur from the alleyway into the orchard trees directly and be less impacted by distance from the floral resources (Table 1).

### 7.1. Natural Enemies

Hedgerows (ranging from 5 m to 57 m in length) adjacent to orchards increased the abundance of rosy apple aphid (*D. plantaginea*) populations, whereas the abundance and duration of *D. plantaginea* decreased with the proximity of flower strips, most likely because natural enemies increased in proximity to flower strips [92]. Hoverfly and ladybird eggs and larvae (Figure 2c) increased in orchards near floral strips, whereas aphid attended by ants significantly decreased with the distance to flower strips. It is hypothesized that by providing alternative sugar resources, flower strips could distract ants from protecting rosy apple aphid attendance and reduce aphid abundance through increased predation by natural enemies [92,93]. 

Natural enemies in cherry trees where wildflower strips were incorporated into the alleyways were not influenced by the proximity to the edge of the orchard, indicating that floral strips had improved natural enemies along the length of the tree row [40,94]. Beneficial insect abundance in blueberry fields was more pronounced in fields adjacent to flower margins, especially in the latter half of the growing season, and natural enemy abundance decreased with increasing distance (0, 20, and 40 m transects) from the field border [95]. Herbivorous insects (species not identified) were also more abundant in fields next to wildflower strips [95].

Spiders and parasitoids in apple and pear orchards declined significantly as distance (0–60 m, 60–120 m, and 120+ m) from the semi-natural habitat increased [96]. The steepest declines were seen when 0 and 120 m into the orchard were compared, but there were no significant declines after 60 m [96]. In Californian vineyards, spider abundance was significantly higher at the vineyard edge than at the furthest distance from the woodland, and abundance was higher at 0 and 50 m into the woodland compared to 50 and 250 m into the vineyard (Hogg and Daane, 2010).

Hoverflies are highly mobile [97], and phacelia pollen was found in the guts of *Melanostoma fasciatum* up to 180 m from the source; a similar trend was observed with *Episyrphus balteatus* and *Metasyrphus corollae*, where pollen was recorded up to 200 m [98] and *E. balteatus* up to 250 m from the flower source [99]. Higher numbers of aphidophagous hoverflies are observed in crops adjacent to flower strips, which is probably due to females searching for aphid colonies in which to lay eggs [100]. Aphidophagous hoverflies, which fed on phacelia and buckwheat floral strips, adjacent to broccoli crops dispersed up to 17.5 m from the floral strips, and very few were observed 50 m from the phacelia floral strips [101]. Wratten et al. [102] also captured the highest numbers of hoverflies close to phacelia strips, but they only sampled up to 12.5 m from the flower strip. Lövei et al. [103] trapped hoverflies with phacelia or coriander pollen up to 75 m from the flower source. 

The colonization of foliage-dwelling spiders (Figure 2d) into small, insecticide-free apple orchards from an adjacent deciduous forest revealed that species composition declined up to 50 m into the crop from the forest edge and was intermediate at 10 m [104].

*Dolichogenidea tasmanica*, a parasitoid wasp of the light brown apple moth (*Epiphyas postvittana*) marked with rubidium from feeding on RbCl sprayed buckwheat plants, were trapped up to 30 m away within seven days of release [105]. In strawberry crops, parasitism by *Copidosoma aretas* of the tortricid, *Acleris comariana*, was measured at 1, 6, and 11 m distances from buckwheat flower strips. Caterpillar mortality was highest near the buckwheat plots [106]. However, as there was no parasitism, this was more likely due to spill-over from a range of predators in the margins [106].

### 7.2. Pollinators

Pollinating-insect abundance and species richness were increased closer to the cherry orchard edge, even with florally sown alleyways [40,94]. More isolated floral areas result in lower flower insect visitor richness, visitation rate (except honeybees), and fruit set [107,108]. Hence, crops that are largely wild pollinator-dependent (contrasted with honeybee dependent) require floristic habitats close to the target crop. Areas of flower-rich habitats within 500–1000 m (study from 250–2000 m) improved the richness of hoverflies and bees [109], and there was no evidence of decreasing abundances of bumblebees or hoverflies with increasing distance from flower strips (1–800 m) [44]. Solitary bees, but not bumblebees, declined at a 400 m range from the flower strips (in more complex landscapes), probably because bees are more centrally-placed foragers, returning to a nest, whereas hoverflies, which do not have a nest, may benefit from even small flower strips [44]. 

In orchards not provisioned with wildflower strips, Fountain et al. [110] found no effect of distance from the edge (up to 50 m) on the quality of pears and no consistent difference in the guild of insects visiting at distances from the orchard boundary. However, honeybees, bumblebees, and solitary bees on apple blossoms (Figure 2e) declined in the orchard from the woodland edge (15, 35, 55, 100, and 200 m from edge) in some years, but findings were not consistent and varied between pollinator group and year [111]. Hofmann et al. [112] suggested that flower strips should be within ~150 m of solitary bees’ (Megachilidae) nesting resource. The maximum foraging distances of small (*Hylaeus punctulatissimus*), medium (*Chelostoma rapunculi*), and large (*Hoplitis adunca*) solitary bees was 1100 m, 1275 m, and 1400 m, respectively [113,114]. However, 50% of female *H. punctulatissimus* and *H. adunca* did not forage at distances greater than 100–225 m and 300 m [115], and it is likely that foraging distance decreases with an increasing number of plant species for solitary bees [114].

The abundance of wild pollinators in cider apple orchards was improved up to 100 m from orchard edges with wildflower alleyways; the same effect was not seen for honeybees [57]. In tomato fields, there was a decrease in pollinators at increasing distance into the field (edge compared to 100 m). Uncommon species of native bees were sevenfold more abundant on hedgerow flowers than on flowers at weedy, unmanaged edges with no significant differences observed in syrphid abundance with distance into the fields. Hedgerows also supported honeybees and acted as net exporters of native bees into adjacent crops [116]. 

At larger scales, seminatural habitats had a marginal positive effect on the species richness of hoverflies and wild bees around apple orchards within a radius of 300 m and 500 m, respectively, with flower resources in orchard alleyways supporting honeybees [43]. At the 100 m and 1000 m landscape scale, inter-orchard flora (12 flowering plant species) and cover of floral resources supported pollinator species’ richness to cherry blossoms [53]. 

In a meta-analysis of 23 studies, including 16 crops on five continents, Ricketts et al. [36] examined the relationship between pollination services and distance from natural or semi-natural habitats. Crop visitation rates of wild pollinators declined at increasing distances into crops, dropping to half at 600 m from the natural habitat. Honeybee visits reduced to half after 2170 m from the seminatural habitat. Species’ richness of pollinators declined by half at 1500 m from the seminatural habitat [36]. Mean foraging distances for bumblebees were calculated between 272 and 551 m (worker foraging distances *Bombus terrestris* 551 m, *B. lapidaries* 536 m, *B. ruderatus* 501 m, *B. hortorum* 336 m, and *B. pascuorum* 272 m) [45]. In general, floral resources should be within 500 m of apple orchards for pollination benefits to be realized [43,51,111].

### 7.3. Production

Isolation from the natural habitat was associated with declines in mango pollinators and in mango production (kg of marketable fresh fruit), but the presence of native wildflower areas corrected these declines [52]. In mango, 42% less production was observed at 500 m from the natural habitat and was attributed to both pollinator abundance and diversity [117]. 

In tunnelled cherry, fruit set and consequently production along the tree rows was impacted. Trees closest to the orchard edge developed more cherries, but these fruit were smaller thought to be a consequence of higher numbers of pollinators at the edge of the orchards [40]. Likewise, in other protected crops, stingless bees and honeybees were more abundant at the ends of tunnels, and there were fewer visits to flowers toward the middle of tunnels. Fruit shape was improved in raspberries with greater pollinator abundance, and yield per plant and mean berry weight were positively associated with pollinator abundance and hence lower at the center of tunnels than at the edge for blueberries [118]. 

Reassuringly, most edge effect manipulations appear to elicit repeatable responses [90], hence benefits can be applied across multiple crops. Edges, whether floral margins or other seminatural habitats (Table 1), offer alternative habitats from cropping systems and provide resources not readily available in crops important for life stages, shelter or a hosts (e.g., Bennewicz [119]). The spill-over of organisms from margins is dictated by distance and quality of the margin area [120]. Duelli et al. [120] concluded ‘in cultivated areas a mosaic landscape of small sized crop fields and semi-natural habitats maximizes arthropod diversity and decreases the probability for overall extinction ….’. Hence, in diverse landscapes, colonization by beneficial arthropods is dependent on habitat suitability rather than size or distance from other non-crop habitats [120]. It might also be prudent to consider spill-over in the opposite direction, especially when floral resources in orchards wain or overwintering habitats are sought by natural enemies [91]. 

## 8. Benefits of Floral Resources to Natural Enemies, Pollinators, and Crop Production 

The majority of studies reviewed tested the impact of floral margins with >30 studies incorporating floral plantings into the crop alleyways and understory. 

### 8.1. Natural Enemies

Natural enemies in fruit crops (Table 2): Most studies aimed at pest control in fruit crops using floral interventions were applied to the orchard area and, usually, in the crop alleyways. He et al. [123] reviewed 70 articles on the dietary value of floral resources in supporting predatory arthropods, including their effect on longevity and fecundity. Floral resources significantly increased predator longevity, but the effect varied greatly among plant species. Flowers with more open or exposed nectaries were more likely to prolong predator longevity. The majority demonstrated increases in the numbers of natural enemies but not always a corresponding increase in pest control. Fewer studies have measured benefits to production. In addition, some of the literature reported poor establishment of wildflower sowings, with differences between treated and control plots in floral establishment being low; thus, the resulting impact was not significant (e.g., Bone et al. [59]). Simon et al. [124] reviewed the impacts on natural enemies and subsequent pest control in fruit crops. Plant management was mostly positive (16 cases) or had no-effect (nine cases), but there were five cases that had a negative impact. The magnitude of pest control was not sufficient enough to reduce pesticide use, except where high levels of damage could be tolerated due to no direct effects on fruit damage or yield, e.g., mites and psyllids.

Wheat (*Triticum aestivum*) sown as a cover crop did not increase natural enemies (*Anthocoris* spp. And *Deraeocoris* spp.) important to pear production [125]. However, studies of apples [126] and vines [105], using buckwheat (*F. esculentum*), resulted in higher parasitism levels (34% compared with 20% in unsown plots) of leaf rolling tortricids [126]. 

Prieto-Benítez and Méndez [127] conducted a meta-data analysis on natural enemies focusing on spiders for pest control. They found negative impacts on spider species richness and abundance for the ten land management types identified (e.g., agroecosystem, plantation, grazing, logging, etc.), except for forests.

Spiders [29,68,96,128] and parasitoids [49,96] benefit from the introduction of floral strips in or adjacent to orchards [96], increasing three to seven-fold in one study of apples [47,69], although not always associated with a significant increase in pest control [129]. 

Bugg and Waddington [33] focused their studies on natural enemies of the codling moth in fruit crops, including tree nuts, pome fruits, stone fruits, and citrus, and they concluded that the parasitism of the codling moth and tortrix larvae was significantly greater under trees with a rich flowering understory. Markó et al. [49] also observed no effect of florally-enhanced ground cover on the codling moth (*C. pomonella*) and the summer fruit tortrix moth (*Adoxophyes orana*) fruit injury. Codling moth infested 36% of apples in organic systems with a cover crop of bell beans (*Vicia faba*), compared to 45% in clean-cultivated organic orchards [29]. Fewer codling moth larvae and damaged fruits were recorded in orchards with wildflower alleyways compared to mown alleyway plots (Fountain et al. unpublished, [50]). However, when deploying codling moth egg sentinel cards, higher predation was observed in short grass cover plots compared to tall grass plots (66% vs. 38%, respectively) later in the season (July and August) [130]. This was attributed to taller vegetation pulling natural enemies away from the trees; there was a higher abundance of earwigs (*Forficula pubescens*) in the short grass plots and no impact of the treatments on spiders or *F. auricularia* [130]. Hence, simply allowing native alleyway flora to grow (‘tumbledown’) does not benefit natural enemies compared to purposely selected floral alleyway sowings. Similarly, encouraging natural flora (wild carrot, parsnip, hogweed, and many other species) in cider orchards did not improve the control of the fruit tree red spider mite (*Panonychus ulmi*), apple pigmy (*Stigmella malella*), aphids, or summer fruit tortrix (*A. orana*) [64]. 

An early study on the parasitism of codling moth larvae found an increase in parasitism from 7% to 34% where nectar-rich flora was implemented [131]. Floral alleyways (in a one-year study) increased codling moth parasitoids [129], while alleyways provisioned with buckwheat in one of two vineyards increased parasitism of leafroller species by >50% [132]. Adult *Anagrus*, sometimes used as a biocontrol agent, were more abundant within the edge of vines sown with buckwheat compared to vines sown with clover (*Trifolium repens*) or mown cock’s-foot (*Dactylis glomerata*), especially early in the season [133]. In addition, parasitism of ‘sentinel’ leafhopper eggs was higher on vines with buckwheat, and parasitism by *Anagrus* of leafhopper eggs on grapes was greater when adults had access to flowering buckwheat rather than buckwheat without flowers [133]. Leafhoppers were not influenced by the species of cover crops used in the same study [133]; this may be important given the future threat *Xylella* transmitted by some species of leaf hopper. Buckwheat is also a host of *Xylella fastidiosa*, which can be transmitted to grapevines [134]. Rates of parasitism of released light brown apple moth larvae (*E. postvittana*) by *Dolichogenidea tasmanica* were higher in areas sown with buckwheat and alyssum compared to phacelia and controls; consequently, leafroller damage was almost 29% lower in floral understorey treatments compared with controls [135]. There were twice as many *D. tasmanica* cocoons in the alyssum and buckwheat treatments compared to the controls [135]. Encouragingly, the parasitoid (*Anacharis zealandica*) of the brown lacewing (natural enemy) was not enhanced by the under-sowings [126,135]. 

Six predator taxa consumed light brown apple moths on ground with cover (*T. repens* and *D. glomerata*), whereas only earwigs (Figure 2f) consumed leafrollers in the vine canopy (×10 activity) [136]. On vines, whilst leafhopper and thrips populations were not influenced by ground cover, the European grapevine moth (*Lobesia botrana*) was also always higher in tilled plots compared to native natural ground cover. However, the vine mealybug (*Planococcus ficus*) was twice as abundant in vines with a cover crop compared to tilled areas; probably because ants, which protect the mealybugs from their natural enemies, were more abundant in these plots (28% vs. 12% of bunches damaged, [137]).

Employing more diverse floral alleyways, spider numbers and their webs increased in apple and cherry trees, reducing numbers of aphids able to return from their summer host plants [40,67,68]. However, an increase in webs is not always mirrored by an increase in web-building spider families (web builders (Theridiidae) and orb web builders (mainly Araneidae)), but species richness of spiders is increased and numbers of jumping spiders (Salticidae) benefit from more complex vegetation cover [68]. Of the total 11 families identified in alleyways and trees, Linyphiidae, Theridiidae, and Araneidae are the most abundant on apple and cherry trees [40,48,138]. Individuals of these families use webs to catch prey, while, Lycosidae, a ground-dwelling spider only recorded in alleyway vegetation, is an active predator [139]. It is likely that dense and diverse vegetation in alleys provide more abundant and diverse prey, including leafhoppers, herbivorous beetles, dipterans, mirids, and thrips [69,95]. Alternative prey can enhance spider abundance and species richness in the canopy of apple trees [69] and help to buffer natural enemies from the effects of disturbance in the crop [95]. 

Floral strips increase the abundance of beneficial insects, particularly later in the season [95], providing late season natural control. In citrus orchards, the ground cover of managed flower mixes enhanced the numbers of spiders, parasitoid wasps, ladybirds, and lacewings in the tree canopy in comparison to plots with bare soil [140]. Cover crops in organic apples also increased the abundance of spiders, parasitic wasps, and ladybirds in the adjacent trees [29]. 

Aphids (e.g., *D. plantaginea*, *A. pomi*) were less abundant in apple trees where floral strips or cover crops were sown [29] in orchards where the numbers of natural enemies (Anthocoridae, Miridae, Namidae, Crysopidae, and Coccinellidae) were generally increased [66,141]. However, Markó et al. [69] found no evidence that habitat diversification enhanced the biological control of the green apple aphid (*Aphis* spp.). Cahenzli et al. [50] demonstrated a slower *D. plantaginea* population increase as compared to standard orchard vegetation, resulting in reduced fruit damage after the second fruit drop. This was coupled with higher numbers of natural enemies in *D. plantaginea* colonies on trees associated with flower strips [50]. In the spring assessments of apple shoots, the abundance of aphids was significantly lower in one year where floral strips were sown in the alleyways compared to unsown and mown alleyways (Fountain et al., unpublished). For cherries, natural enemies increased by 73.9% and 12.9% in alleyways and trees, respectively, compared to the growers’ standard grass alleyways. As a result, aphid removal from sentinel cards was 25.3% greater in cherry trees adjacent to wildflower strips compared to controls [40]. Higher densities of web-building spiders in orchard plots with wildflowers reduced winged aphids returning from their summer host plants, resulting in fewer *D. plantaginea* in the trees the following spring [67]. Although Vogt and Weigel [142] did not see an impact of flora on *D. plantaginea* on the trees, there was a suppression effect of the green apple aphid (*A. pomi*). *D. plantaginea* and ants were also less abundant in cider apple trees near the flower margins, which also favored natural enemies [92].

**Figure 3:**
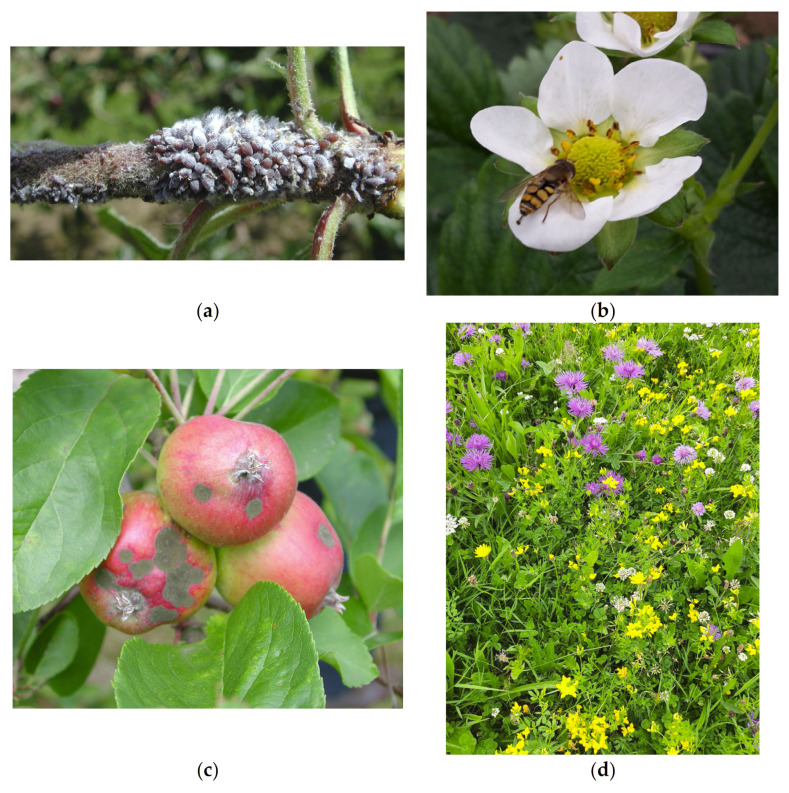

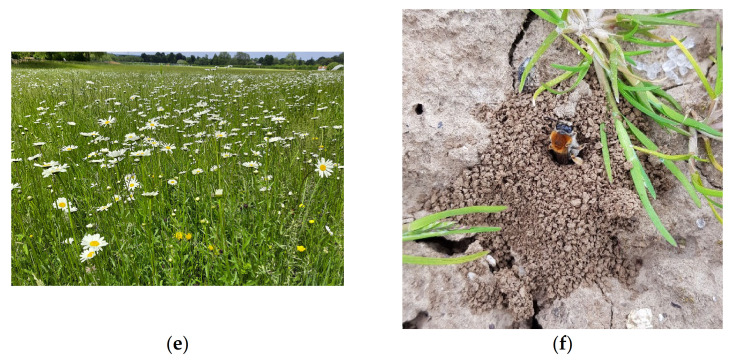
(**a**) Woolly apple aphid colony (credit NIAB EMR), (**b**) hoverfly on a strawberry flower (credit NIAB EMR), (**c**) apple scab (*Venturia inaequalis*) on damaged fruits (credit NIAB EMR), (**d**) diverse and abundant floral mix of different flower types (credit Celine Silva), (**e**) oxeye daisy (*Leucanthemum vulgare*) (credit Celine Silva), and(**f**) solitary bee nest with exposed tumuli (excavated soil) above ground (credit Konstantinos Tsiolis).

Faster suppression of the woolly apple aphid (*Eriosoma lanigerum*) (Figure 3a) occurred on apple trees closer to sweet alyssum flowers compared to mowed grass. Higher densities of natural enemies were also observed near sweet alyssum plantings and found to move between alyssum and adjacent apple trees [143]. 

The abundance and diversity of predatory phytoseiid mites increased with flowering ground cover in the spring and autumn, preventing a build-up of spider mite [49]. However, single species’ sowings of 14 different flowering plants did not affect fruit tree red spider mite (*P. ulmi*) abundance in trees [144]. 

Encouragingly, the European tarnished plant bug (*Lygus rugulipennis*) was less abundant in sown flowers compared to the control (regularly mown) plots, and cockchafers (*M. melolontha*) were less abundant in the floral compared to bare ground plots [69]. Bostanian et al. [47] also observed less damage by European tarnished plant bugs and summer tortricids in florally-managed apple plots compared to the conventionally-managed controls. Conversely, ground cover that included wild carrot, parsnip, and hogweed favoured the common green capsid (*Lygocoris pabulinus*) [64].

Lacewing adults (*Chrysoperla carnea*) were also more abundant where flower mixes were established [69], and coriander planted in strawberry crops increased lacewing egg laying in aphid colonies [145]. 

In a pear orchard study by Winkler et al. [146], numbers of anthocorids in adjacent pear trees were initially significantly higher in floral (*Centaurea cyanus*, *Fagopyrum esculentum*, *Lobularia maritima*, *Thymus serphyllum*, and *Sinapis alba*) than in control plots. In this study, it was not possible to detect an impact on the control of the pear sucker (*Cacopsylla pyri*) because management (including reduced pesticide use) meant that the pear sucker declined in the control equally well to the florally-treated areas [146]. In semi-field experiments with a single species of flowering plants around pear trees, anthocorid numbers were boosted by corn chamomile and cornflower, and seasonal totals of anthocorids were higher in the under-sown trees with floral provision than the bare earth plots [144]. Although none of the 14 individual sown species in this experiment affected the abundance of the pear sucker (*Cacopsylla pyricola*), numbers of psyllid larvae did decline more quickly on the trees surrounded by flowering plants [144]. Alleyway floral sowings in organic pear orchards decreased suckers (*Psylla chinensis*), aphids (*Aphis citricola*), and mealybugs (*Pseudococcus comstocki*), and in some cases, it delayed their establishment [65]. In pear orchards, natural ground cover and sown ground cover (*Lolium perenne*, white mustard, *Sinapis alba*, and white clover *T. repens*) also sheltered distinct arthropod communities, with the former characterized by spiders and the sown ground cover characterized by ants. Anthocoridae (Heteroptera) and Miridae (Heteroptera) were the main beneficial insects on pear trees in sown areas with Empididae (Diptera) and Miridae more abundant in the natural ground cover area, and earwigs and Miridae more abundant in bare ground areas [147].

The provision of floral strips usually has a positive effect on hoverflies [47,48,95]. In experiments screening fourteen flowering plant species, ladybirds were particularly abundant on cornflowers [144]. The impact of floral margins can also vary between years, with increases in hoverflies and lacewings not being evident every year (Fountain et al. unpublished). In protected cherry orchards, flower sowings in alleyways had greater pest regulation services (measured using aphid baited cards) compared to regularly mown, predominantly grass alleyways (by 25.3%). Natural enemies increased by 73.9% and 12.9% in alleyways and trees, respectively, compared to the conventional control [40]. Numbers of natural enemies (*Coccinella septempunctata*, *Phytoseiulus persimilis*, and *Chrysoperla sinica*) increased in organic pear orchards with alleyway sowings of the aromatic plants summer savory (*S. hortensis*), ageratum (*A. houstonianum*), and basil (*O. basilicum*), with the ratio of natural enemies to pests being higher in orchards with inter-row plantings [65]. More Ichneumonoidea and hoverflies were observed in floral experimental blocks (*Tanacetum vulgare*, *Chrysanthemum maximum*, *Aster tongolensis*, and *Achillea millefolium*) than untreated control blocks in apple orchards, with no increase in damage by key pests compared to the control plots in a five-year study [47]. Sweet alyssum flowers are also attractive to hoverflies [143]. 

**Figure 4:**
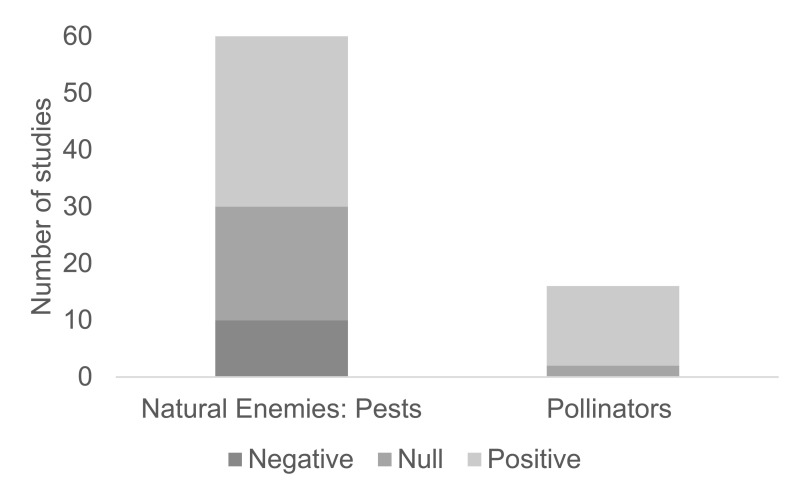
Summary of the studies in Table 2 and Table 3 showing the numbers of floral enhancement studies in fruit crops that recorded negative, null, or positive impacts on fruit pests and/or their natural enemies (densities, fruit damage, number of pesticide applications), and on pollinators, including fruit production. NB: data not statistically analyzed.

In general, crops that have enhanced ground cover have lower pest levels, a greater number of species with higher abundance of predaceous arthropods, and higher removal rates of artificially-placed prey (Figure 4) compared to crops that have florally impoverished ground cover [29]. 

Nevertheless, it is unlikely that key pests will be sufficiently controlled by floral interventions to a commercially acceptable level. For example, the codling moth has a very low threshold because one caterpillar can render a single fruit unmarketable. However, for pests that do not directly damage fruits and cause superficial damage to foliage, for example, floral margins can boost local levels of natural enemies, which negate the need for some insecticide applications [35]. This strategy will rely greatly on regular and accurate pest scouting, monitoring, tracking, and reporting. 

### 8.2. Pollinators

Pollinators in fruit crops (Table 3): Most fruit crops are highly dependent on insect pollinators [54]. Pollinator diversity is higher in fruit crop landscapes containing hedgerows, meadows, and suburban areas, as these provide nesting and floral resources throughout the spring and summer for species that are reliant on resources beyond the crop area [170]. Because most fruit-pollinating bees are generalist species, promoting floral resources around the farm and landscape will help to sustain diverse wild bee populations for fruit crop pollination [170]. Apple seed set was increased, shape was improved, and pollen limitation decreased if wild bee species’ richness and abundance were increased, resulting is less reliance on honeybees [171,172]. Nicholls and Altieri [1] reviewed the impact of a semi-natural habitat in agriculture on pollinators and identified that some weed species were important pollen and nectar sources. Hence, weed levels below economic impact should be tolerated to support pollinators. Areas of intensive farming, field margins, field edges and paths, headlands, fence-lines, rights of way, and nearby uncultivated patches of land are pollinator refuges that could be optimized for pollinators with appropriate management. A meta-analysis of 109 studies found that most insect pollinator groups responded positively to increasing plant species richness, but plant selection was key to support agroecosystems and improve biodiversity [73].

Ratios of pollinator groups visiting fruit crops varies; the ratios of honeybees to wild bees in apples, pears, blueberries, and raspberries, for example, was 10:1, 2:1, 1:5, and 5:1, respectively [110,170], with Andrenid, ground nesting bees, the most abundant of the wild bees visiting apple and pear flowers [110,170,173].

Although honeybee abundance remained static, three and four years after sowing wildflower plantings adjacent to blueberry crops, wild bee and hoverfly numbers increased [62]. However, orchard ground cover is crucial in supporting honeybees in apple orchards, with wild bee visitation increasing with the proportion of high-diversity bee habitats in the surrounding landscape (1 km radius) [43]. Orchard mason bee (*Osmia lignaria*) nests installed in areas adjacent to apple orchards were more successful if they had access to sowings of bigleaf lupine (*Lupinus polyphyllus*) [167]. 

Cider apple orchards with alleyway wildflowers increased wild bee and Diptera visits to apple flowers by 40% [57]. This effect was more pronounced when the orchards were also next to semi-natural habitats [57]. In a two-year study on blueberries, sour cherries, and watermelon, a 117% greater wild bee abundance, 75% greater richness, and 57% greater diversity in the floral margins did not improve pollinator abundance in the crops [166], suggesting that, in some instances, benefits to the wild bee community gained from enhancements do not spill over into the crops. However, in a large-scale study involving 85 apple orchards, on a landscape gradient, a higher cover of flowering plants within and adjacent to apple trees did increase flower visitation rates by pollinating insects [51]. In commercial dessert apple orchards, although hoverfly diversity and species richness were greater in orchards with wildflower strips, this did not translate to more visits to apple blossoms by any pollinator group, which was attributed to the use of pesticides in the orchards [48]. Pesticide use was also found to be a key contributor to pollinator decline in pigeon pea (*Cajanus cajan*), despite practices to improve pollinator abundance [174], and it resulted in the lower species richness of bumblebees in apple orchards [60]. Orchard management should incorporate the consideration of pollinators into IPM and adopt integrated pollinator-pest management (IPPM) considering the creation of habitats for pollinators, landscape management, and agroecosystem diversification with a move toward better times and selection of softer protection products [175,176].

In polytunnel strawberry crops, the frequency of pollinator visits was 25% higher in crops with adjacent flower strips compared to those without, with a combination of wild and commercial bumblebees accounting for 67% of all pollinators observed [165]. In a three-year study on polytunnel grown cherry, floral alleyway sowings resulted in an increase in pollinating insects in the summer (after the cherry blossom period) with benefits to production [94]. Flowering plants in alleyways of cherry orchards are also a driver of pollinator diversity and abundance and the fruit set of sweet cherry [53]. Although two thirds of all flower visitors to sweet cherries were honeybees, the fruit set was linked to wild bee visitation [54]. However, not all perennial crops benefit from wildflower interventions; for example, cocoa production is reliant on flower visits by ceratopogonid midges, hence the augmentation of ground cover using mulches is needed to increase midges and yield in this crop [177].

It is essential that wildflower habitats are not considered in isolation and are combined with landscape management approaches for pollinators [178,179]. Although they provide food, other landscapes such as woodlands may be needed for nesting [58,168]. Lack of these other habitats is known to be a limiting factor of bee abundance and diversity [180,181]. Where mass flowering crop cover increases in a landscape, the densities of bumblebees, solitary bees, honeybees, and hoverflies (Figure 3b) decrease by 15, 10, 15, and 7%, respectively, creating a diluting effect [182]. In addition, field margins in landscapes with flower strips have higher bumblebee abundances compared to landscapes without flower strips, while farms with higher quality and area of flower strips have more bumblebees and solitary bees in field borders [44]. Pollinators are subject to multiple stressors, including parasites, pesticides, and a lack of resources. Fruit growers can support pollinators by incorporating flower-rich habitats into farmland, reducing pesticide use through adopting more sustainable farming methods, and managing commercially-reared pollinators so that the transmission of parasites and diseases is minimized [183]. Pollinators are more affected by landscape heterogeneity than adjacent field margins [184], and dispersing patches of natural habitat throughout the landscape to create habitat heterogeneity will support higher bee abundance even in landscapes with a low proportion of natural habitat overall [185]. Fruit crops typically bloom for a short period of time and cannot sustain insect pollinators in isolation. Additional floral resources in orchards can provide a greater diversity and abundance of flowering plants before, during, and after blossom to support and attract pollinating insects in and around fruit crops [53]. However, compared to the numbers of studies on pests and natural enemies, there are fewer studies (Figure 4) on a limited number of fruit crops (Table 3), and, hence, more evidence of impacts is advisable before tailored floral resources are installed.

### 8.3. Detrimental Effects

When implementing floral resources, it is essential that flora introduced into perennial crops do not act as alternative hosts or introduce pests, diseases, or storage rots [186]. 

For fruit crop diseases, Hawthorn (*Crataegus* spp.) might be avoided near to pear orchards to reduce the spread of fire blight [147]. White mustard sowing increased russets and reduced the fruit weight of apples [59]. Significantly more disease has also been observed in apple orchards with white clover (*Trifolium repens* L.) cover crops [187], especially postharvest storage rots of apples [188]. In addition, white clover harbors few beneficial insects in comparison with annual clovers (*Trifolium* spp.) and vetches (*Vicia* spp.) [33]. In organic apple orchards, although most impacts were positive, there was more damage to fruit from apple scab (*Venturia inaequalis*) (Figure 3c) in plots with wildflower alleyways than in crops where ‘weeds’ were controlled by mechanical disking [189].

*Lygus* spp. has been detected in some alleyway cover crops in apples and hops [29,190], although damage to fruit was not recorded in these studies. Floral ground cover increased numbers of common green capsid (*L. pabulinus*) in cider apple orchards in the Netherlands [64], but in apple floral strips in Hungary, the European tarnished plant bug (*L. rugulipennis*) was less abundant than in the control treatments [69]. Likewise, damage by the tarnished plant bug was lower in managed plots compared to controls after five years (no insecticides) [47]. In North Carolinian peach orchards, Meagher Jr and Meyer [32] demonstrated that weedy plots dominated by chickweed (*Stellaria media*) and Carolina geranium (*Geranium carolinianum*) had higher percentages of *Lygus* damaged fruits (Table 2). In general, Heteroptera diversity is increased in orchards with floral plots, including predatory species [191]. Killan and Meyer [192] recorded lower cat-facing damage to peaches in herbicide-treated blocks compared to fruit sampled from weedy areas, demonstrating the need to implement the most beneficial flora within orchards. 

The mullein bug (*Campyloma verbasci*) was also observed at higher incidences in florally sown orchards [193] and increased woolly apple aphid infestations in floral treated plots in one trial, but only in the first year after establishment [69]. 

In a two-year study in an experimental apple orchard, two pests, the apple sucker (*Psylla mali*) and the cercopid froghopper (*Philaenus spumarius*), increased in number in flower apple orchard alleyways, although no major damage was observed [141]. The implications of *Xylella fastidiosa* (a bacterial disease of woody species) spread should be considered with increases of *P. spumarius,* which is a vector of the disease.

*C. cyanus* and *L. vulgare* attracted high proportions of thrips (some of which are pests), and *K. arvensis* and *A. millefolium* were attractive to pollen beetles [194]. Plant bugs (Miridae) were also more abundant in fields with flowering plant strips, as were plant hoppers (suborder Auchenorrhyncha) and thrips [95]. However, the latter study did not relate the impact of the floral resource to crop damage, and it is not known if these areas act as a sink, a source of pests, or indeed a combination of these factors. Hence, phytophagous insects are generally increased in areas treated with floral interventions, but these are primarily non-pest species and serve as alternative prey for natural enemies [195]. This is useful in periods when crop pest abundance is low, e.g., earlier in the season.

Albert et al. [92] found that the only significant negative effect of hedgerows in the vicinity of cider apple orchards was a decrease in the presence of ladybird larvae in the orchard. However higher numbers of hoverfly larvae and eggs were found in the crop adjacent to hedgerows. 

Some negative impacts of floral alleyways have been attributed to an increase in spider mite (e.g., Campbell [190]); however, this is often accompanied by increases in the natural biocontrol, phytoseiids (predatory mites). 

Grapevine vigor was reportedly lower with cover crops, compared to no-cover crop alleys [156]. Terminal growth was particularly depressed for apple trees with understories of white clover and grass [196]. In addition, in vines, native natural ground cover (compared to tilled areas) had more abundant populations of ants, which protected mealybugs from natural enemies [137]. Consequently, a reliance on the resident colonization of flora may not deliver pest control benefits and may enhance pests.

Leafrollers (e.g., *E. postvittana*) had an increased longevity and egg production fecundity in the presence of alyssum (*L. maritima*) [135]. Flower margins may also be suitable habitats for slugs creating a microclimate refugia [197,198,199]. In orchards where docks have been allowed to grow, dock sawfly (*Ametastegia glabrata*) can move onto developing apples (pers. obs.), causing fruit damage [200]. Alternative host species for other pests, such as plantain (Plantaginaceae) for rosy apple aphid (*Dysaphis plantaginea*), should be avoided. More research is needed to determine the risk of additional pest pressure from these species. 

The abundance of earwigs (*Forficula pubescens*) was positively correlated with codling moth egg predation in regularly-mown plots, but it was negatively correlated in orchard plots where grass was left to grow [130]. The authors of this study suggest that earwigs might find an alternative resource in the taller grass and that growers could mow at key times in the season to increase the foraging of codling moth [130]. This could be predicted using codling moth flight pheromone traps and temperature-based models (e.g., RIMPro). Flower mixes in orchards may change the community composition of invertebrates; for example, in a study by Markó and Keresztes [68], the dominance of one spider species resulted in a lower overall spider diversity. 

Another potential detrimental effect of floral margin implementation is the distraction of managed pollinators (e.g., honeybees) from pollinating crops. In Scottish raspberry crops, commercially produced bumblebees had 12% and 15% pollen from *Rubus* and *Potentilla*, respectively. The remaining pollen on the bees was from non-target wildflowers [201]. However, this study did not measure the impact on crop pollination. Another study demonstrated the potential to divert managed bees to crops with the use of caffeine coupled with a reward and the odor of the focus crop [202]. In spring crops of open-ended polytunnel grown strawberries provisioned with bumblebees, there was a significant increase in marketable yield compared to strawberries without bumblebee provision [203]. In spring blossoming tree fruit, most sown wildflowers would not be flowering and not encourage competition. However, more studies would be useful to determine the impacts of early wildflower distraction, e.g., dandelion, during pome and stone fruit blossom. 

The effects of floral margins can also be inconsistent [190] with interannual differences of the benefits the crops receive. However, these may be transient negative effects, especially in the establishment year, until beneficial insects have established and built in abundance. Careful selection of plants is important to avoid any risk of enhancing pest populations or offering an alternate host for plant pathogens and other noxious organisms. Ideally, plants should be botanically unrelated to the crop [204].

## 9. Choice of Floral Resources

Guidance is increasingly available on the choice, establishment, and management of wildflower strips in and around crops (e.g., Nowakowski and Pywell [205]; https://northsearegion.eu/beespoke/publications-downloads/ (accessed on 17 March 2022)). To maintain the ecosystem services provided by insect pollinators and natural enemies, a diverse mix of species and functional groups of flowering plants are needed [206]. Planting areas are recommended to be at least 3–10 m in width and can be selected for sowing on a range of soils [204]. They should form part of an ecological intensification approach that aims to regulate, support, and even increase crop production [63]. Sowing orchards with field margins is complicated by restricted space and continuous travel by vehicles on headlands. However inter-row (alleyway) sowings may be beneficial, particularly if orchards are too large to allow natural enemies to penetrate [29]. It may even be possible to adjust wildflower mixtures with aromatic plants such as summer savory (*S. hortensis*), ageratum (*A. houstonianum*), and basil (*O. basilicum*), to repel specific pests [65]. In addition, the area available from alleyways for floral resource far outweighs that of the orchard perimeter, ensuring food and shelter for pollinators and natural enemies throughout most of the fruit-growing season. Sown wildflower strips (Figure 3d) support higher insect abundances and diversity than cropped habitats, especially pollen- and nectar-rich flower mixtures [207]. Although common insect species are the main beneficiaries of agri-environmental schemes [207], there is the potential to optimize floral mixes, depending on the service required, and increase the number of wildflowers at a landscape scale to increase their overall effectiveness [208].

To promote natural enemies and pollinators on farms, land managers should aim to: (1) identify where they already have sources of good quality flora and protect these areas, (2) enhance and improve areas that are adequate, but not giving the best service, (3) connect areas of floral resource (e.g., hedgerows, woodlands, and/or meadows) by creating corridors to enable beneficial insects to move around the landscape [83], and, (4) create new areas of floral resource on farm areas lacking heterogeneity. Habitat manipulations should be coupled with the complete lifecycle requirements of the beneficial insects, including nesting, overwintering, and breeding sites. 

Orchards are generally devoid of flowers post-bloom but need to support insects though the growing season. Because bee diversity is related both to flower cover and diversity [209], choosing a floral mix with functional diversity (components of biodiversity that influence how an ecosystem operates or functions) should be considered to encourage higher species diversity and deliver more ecosystem services [210]. By increasing both plant species’ richness and abundance, flower visits by bees will be promoted [211]. 

Floral mixes can be manipulated according to floral traits (Figure 3d) to target the ‘types’ of beneficial insects required [212]. To provide for insects with short mouthparts, forage with easily-accessible nectar, particularly Asteraceae, Umbelliferae, and Fabaceae are beneficial [213]. For example, flowers of buckwheat (*F. esculentum*), cornflower (*C. cyanus*), alyssum (*L. maritima*), coriander (*Coriandrum sativum*), mint (*Mentha spicata*), yarrow (*A. millefolium*), Phacelia (*Phacelia tanacetifolia*), fennel (*Foeniculum vulgare*), Korean liquorice mint (*Agastache rugosa*), wild parsnip (*P. sativa*), corn marigold (*Chrysanthemum segetum*), borage (*Borago officinalis*), wild carrot (*Daucus carota*), hairy white oldfield aster (*Aster pilosus*), chamomile (*Matricaria recutita*), mallow (*Malva sylvestris*), cow parsnip (*Heracleum maximum*), and vetch (*Vicia sativa*) are attractive to adult hoverflies [97,101,213,214,215,216,217,218,219]. Buckwheat and phacelia are sucrose-rich [135,220,221,222], while borage has a high nectar production [223]. 

Nectar availability can be limiting for parasitoids [132], and so flowers with open nectaries are important [224]. This was achieved with planting creeping cinquefoil (*Potentilla reptans*), yarrow (*A. millefolium*), white clover (*Trifolium repens*), common hedge parsley (*Torilis arvensis*) [129], corn marigold (*C. segetum*), and corn chamomile (*Anthemis arvensis*) [144].

Yarrow (*A. millefolium*) and oxeye daisy (*Leucanthemum vulgare*) (Figure 3e) attract multiple beneficial arthropods [225]. Perennial stinging nettle (*Urtica dioica*) is a reservoir of natural enemies, including pirate bugs (Anthocoridae), Miridae, and ladybirds (Coccinellidae) [226,227]. Anthocorids are also abundant on cornflower (*Centaurea cyanus*) and corn chamomile (*A. arvensis*) [144]. 

Scabious (*Knautia*), knapweed (*Centaurea*), and thistles (*Cirsium*) are regularly visited by bumblebees and butterflies [228]. Knapweed (*Centaurea*) flower coverage also has a strong positive effect on crop pollination services [178]. Including a range of Umbelliferae, Asteraceae, and Geraniaceae in seed mixes caters to a wide diversity of bee species, with 14 wildflower species across nine families attracting 37 out of the 40 bee species recorded in a farm study [229]. Kidney vetch (*Anthyllis vulneraria*) and meadow cranesbill (*Geranium pratense*) were highly attractive to bumblebees, and smooth hawk’s-beard (*Crepis capillaris*), wild mustard (*Sinapsis arvensis*), field bindweed (*Convolvulus arvensis*), and rough chervil (*Chaerophyllum temulum*) were attractive to solitary bees [229].

For higher blossom visit frequency to apple orchards, floral mixes should be tailored towards species preferred by andrenid bees [57,70,230]. Evidence suggests that dandelions also enhance andrenid bees, so they can be managed in alleyways as an early flowering resource [57,169]. Spring flowers (March to May) are vitally important for nest-founding bees (Figure 3f). 

Flower density is also a good predictor of insect diversity [231], so growers may consider minimizing the ratio of grasses to flora where possible. In addition, some non-native species are capable of extending the flowering season [232]. Growers might also consider adding to floral mixes species that flower more consistently, e.g., clovers (*Trifolium hybridum, T. pratense, T. repens*, *L. corniculatus*), cornflower (*C. montana*), vetches (e.g., *V. cracca, V. sativa*), and wild carrot (*D. carota*) [121]. Legumes are particularly important for bumblebees (but species with a shorter corolla can be selected to encourage shorter proboscis insects, e.g., hoverflies) [233,234], in addition to providing a source of nitrogen to orchards [235]. In addition, tall grasses are needed for overwintering bumblebees [205].

## 10. Establishment and Management of Floral Resource

To establish a perennial wildflower area, ensure that the seedbed is firm, fine, and weed free and sow the seeds on the surface of the soil, then broadcast [205]. Ideally, wildflowers should be grown with season-longevity in mind. More details on how to establish wildflowers successfully can be found in Nowakowski and Pywell [205] and https://www.silenceofthebees.eu/wp-content/uploads/2021/06/BEESPOKE-Establishing-Perennial-Wildflowers-Leaflet-WEB.pdf (accessed on 17 March 2022). Floristic species composition should be selected with soil conditions [236] and establishment in mind (e.g., herbicide applications) [33,150]. 

**Figure 5:**
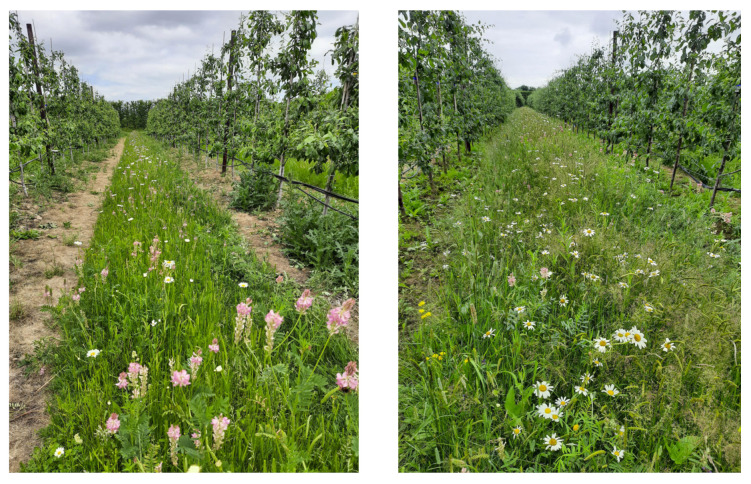
Orchard alleyway sowings with diverse flora and structure for natural enemies and pollinators (credit Celine Silva).

Plant size influences the number of beneficial insects visiting floral resources; for example, increasing the plant size of brassicas increased the species richness of insect herbivores, natural enemies, and pollinating insects. Plant heights from 10 to 130 cm led to a 2.7-fold increase in predicted total arthropod species richness [237], and repeated mowing can have a negative impact on wild bees [78]. Hence, reducing mowing regimes and applying fewer applications of herbicides will encourage flowering species [57] and web-building spiders [238]. The number of aphids per spider web decreased with increasing management intensity from 8.5 ± 4.0 (mean ± SE) aphids at uncut sites to 4.7 ± 1.8 aphids at sites that were managed by cutting [239]. Reducing mowing regimes from 2–3 to only once per month increased the numbers of predators and parasitoids in pear orchards because of an increase in food resources, e.g., non-pest aphids, *Lygus* spp., and leafhoppers/planthoppers [239]. Numbers of spiders and a predatory mirid, *Deraeocoris brevis,* were also higher in fruit trees where the ground flora was only mowed once per month [239]. Increasing sward architecture can increase the total biomass of invertebrates by around 60%, providing food for higher trophic levels, such as birds and mammals [71]. There was no difference in natural enemy abundance, richness, or pest control when these were recorded and compared in two wildflower management regimes; a standard single cut in late September or regularly cutting to a height of 20 cm throughout the growing season in cherry alleyways. However, the numbers of predators in the cherry trees were 15% higher compared to standard regularly-mown grass alleyways [40]. Cutting half the margin mid-season will also prolong the floral resources that are available [205]. Some mowing is required to preserve vegetative and flower buds and permit regrowth [33,40]. Other management practices to consider in orchard alleyway sowings (Figure 5) include cutting every other row on a rotation [210] or selecting floral areas at different stages of succession and/or with different plants to provide habitats for various insect groups [207] and seasonal continuity. To reduce costs, seed mixtures can have a simple composition if key plant species are provided [240]; however, wildflower strips may need to be resown if flowers begin to decrease [241]. 

Wildflower areas will provide (1) alternative prey or hosts when pests become temporarily scarce, (2) alternative food sources such as nectar and pollen for adult predators and parasitoids, and (3) shelter or undisturbed habitats as refuges and overwintering sites [242,243]. However, unmanaged strips have the potential to shelter rodent pests, which without control could become a severe problem in orchards [244]. 

## 11. Overall Conclusions and Future Directions

This review focused on the benefits that additional floral resources in the vicinity of fruit crops provide to pest regulation and pollination services through the provision of natural enemies (predators and parasitoids) and pollinating insects. Fruit orchards with flowering ground cover contribute to pest management and pollination by boosting beneficial insects with variable- and context-dependent outcomes. The effect of floral resource provision depends greatly on the quality of floral resource provided, location, landscape, pesticide use, and management of the floral resource. For example, in a depauperate landscape, there will not be the beneficial insects to utilize the floral resource, and where there is ‘over-use’ of pesticides, any positive impacts will be negated.

The economic and production impacts of floral resources on fruit crops are largely under-studied in most of the literature, and the duration of most studies (less than 3 years) does not give time for populations or the diversity of many beneficial arthropod groups to respond. However, the longer-term studies do mostly demonstrate positive impacts on beneficial species, even if this does not always equate to improved production and economic benefits. Floral resources generally need to be within 500 m of the target crop, but arthropod fauna respond according to size, ability to distribute, and whether they are reliant upon returning to a nest site. 

Overall, the impact of wildflower sowings on crop production is either benign or positive with either low or under-reported negative impacts (Table 2 and Table 3, Figure 4) [13]. This review highlights the isolated nature and need for larger-scale studies to tailor flowering resources to specific crops and landscapes to further advance the science and benefit to fruit growers. 

Many studies do not consider the dual nature of insects, e.g., pollination and predation from hoverflies [219], or studies are conducted in a restricted time period [63,245] or do not follow through to economic impacts, including yields and costs of providing floral resources [246]. Profitability is primarily driven by spillover of beneficial organisms contributing to biocontrol [122,178,247,248] and pollination, and a higher focus on economic gains would encourage take-up of diversifying agroecosystems [245].

However, scientists need to work closely with growers to understand the specific requirements of growing systems and the potential negative impacts on the business that implementing and maintaining floral resources might have. Often, fruit growers do not have the time or resources to invest in understanding or implementing such changes and rely on agronomists for advice and evidence of new practices. This shifts the emphasis one step from the grower, making them removed from the benefits that floral provision may provide. Tools for floral establishment and long-term maintenance are also needed alongside long-term monitoring so that habitats can be adjusted to the requirements of the crop. 

However, there is ample evidence that provisioning florally-diverse areas with long-lasting floral resources through the season [72] provides resources to beneficial insects [182,249,250]. Wildflower areas increase the predator to prey ratio in crops [213], and designing agricultural areas that integrate land use and ecosystem function is a practical approach for promoting sustainable agriculture practices [251] and promoting less interannual variability between beneficial arthropod populations [75]. 

Areas of species-rich and abundant floral resources [72,209,252] provide food (pollen nectar, nectar, vegetation, and prey [97]), nesting sites, structure to build, e.g., spiders webs, [253] and an area of refuge in poor weather and for diapause during the winter. These areas can be kept pesticide free [174] through positioning or through the targeted use of precision agriculture [48] and can even impact arthropod abundances in the wider landscape [44]. 

Fruit crops lend themselves to floral resource provisions [77] because they are grown in rows (e.g., tree fruit [49,50]) or elevated structures (e.g., table-top strawberries), allowing alleyways and understories to be sown to benefit the crop (Figure 5).

However, floral resources should be planned with the landscape in mind [164,179], while being in the proximity of the crops (Table 1). In addition, floral resources should not be implemented without considering the preservation of semi-natural areas in the landscape, which are key to provisioning the full life cycles of many insects [82,254]. Floral resources should not be implemented in isolation of other beneficial insect needs and can be enhanced by the proximity of existing features [255] and inter-connectedness of well-managed habitats that have complementary resources [256].

Future long-term studies of floral resources in fruit crops should tailor floral mixes to specific crops to provide the highest benefit, while reducing any negative impacts (e.g., introduced pests, diseases, or undesirable microclimate). 

## Figures and Tables

**Table 1:** Distance of spill-over of pollinators and natural enemies from floral resources and the seminatural habitat of importance to fruit crops.

Group	Measure	Distance into Crop	Author
Pollinators			
Pollinators	Species richness	Halved at 1500 m	[36]
Pollinators	Abundance	Up to 100 m	[116]
Wild pollinators	Abundance	Up to 100 m	[121]
Wild pollinators	Visitation rates	Halved at 600 m	[36]
Honeybees	Visitation rates	Halved at 2170 m	[36]
Honeybees, bumblebees, and solitary bees	Abundance	Declined 15–200 m	[111]
Solitary bees	Abundance	400 m	[44]
Solitary bee; *Hylaeus punctulatissimus*	Foraging distance	Halved at 100–225 m	[115]
*Hoplitis adunca*	Foraging distance	Halved at 300 m	[115]
Bumblebee worker; *Bombus terrestris*	Foraging distance	551 m	[45]
* B. lapidaries*	Foraging distance	536 m	[45]
* B. ruderatus*	Foraging distance	501 m	[45]
* B. hortorum*	Foraging distance	336 m	[45]
* B. pascuorum*	Foraging distance	272 m	[45]
Bumblebees or hoverflies	Abundance	>800 m	[44]
Hoverflies and bees	Richness	500–1000 m	[109]
Hoverflies	Abundance	>100 m	[116]
Hoverflies	Abundance	At least 12.5 m	[102]
Hoverflies		Up to 75 m	[103]
Hoverfly; *Melanostoma fasciatum*	Presence	180 m	[98]
* Episyrphus balteatus* and *Metasyrphus corollae*	Presence	200 m	[98]
**Natural enemies**			
Spiders	Abundance	0 and 50 m	[122]
Natural enemies	Decreasing abundance	0, 20 and 40 m	[95]
Spiders and parasitoids	Abundance	up to 60 m	[96]
Aphidophagous hoverflies	Presence	17.5 m	[101]
Spiders (foliage dwelling)	Species composition	~10 m	[104]
Parasitoid wasp; *Dolichogenidea tasmanica*	Presence	Up to 30 m	[104]

**Table 2:** Effects of wildflower of cover crop floral enhancements on the control of fruit pests, updated from Simon et al. [124] ¹ and updates ². The effect of plant manipulation on pest control is considered to be positive, null, or negative when either the density of the pest arthropod of the fruit tree, fruit damage, and/or the number of pesticide applications against the target pest is lower, equal to, or higher, respectively, compared with the control. NB: different effects may be due to species growth, location, or timing.

Fruit Crop	Pest Group	Target Pest(s)	Plant Manipulation(s) or Presence	Effect on Pest Control	Source
^1^ Apple	Aphid	*Dysaphis plantaginea*	Flower strips	Negative	[142,148]
^1^ Peach	Hemiptera	Leafhoppers	Plant cover	Negative	[149]
^1^ Peach	Hemiptera	Hemiptera species	Plant cover	Negative	[32]
^2^ Apple	Heteroptera	*Lygus*	Flower plant mixture, alleyways	Negative	[29]
^2^ Apple	Heteroptera	*Lygocoris pabulinus*	Flowering weeds, alleyways	Negative	[64]
^2^ Pear	Heteroptera	*Lygus*	Cover crops, wheat	Negative	[125]
^2^ Apple	Homoptera	*Eriosoma lanigerum*	Flower plant mixture, alleyways	Negative	[69]
^1^ Peach	Spider mites	*Tetranychus urticae*	Plant cover	Negative	[150]
^2^ Vines	General	Various	Buckwheat	Negative	[134]
^1^ Apple	Aphid	Apple aphids	Peach nectaries	Null	[151]
^1^ Apple	Aphid	*Aphis spiraecola*	Buckwheat	Null	[151]
^1^ Apple	Aphid	*Aphis pomi*	Flower strips	Null	[142,148]
^2^ Vines	Cicadellidae	Leafhoppers	Buckwheat, alleyways	Null	[133]
^2^ Apple	General	Various	Flowering weeds, alleyways	Null	[64]
^1^ Apple	General	Apple pests	Plant cover	Null	[152]
^2^ Apple	General	Various	Flower plant mixture, alleyways	Null	[68]
^2^ Apple	General	Various	Flower plant mixture, alleyways	Null	[48]
^2^ Apple	Homoptera	Green apple aphids (*Aphis* spp.)	Flower plant mixture, alleyways	Null	[69]
^2^ Apple	Lepidoptera	Codling moth	Flower plant mixture	Null	[129]
^1^ Apple	Lepidoptera	Tortricidae	Phacelia	Null	[135]
^2^ Pear	Psyllid	*Cacopsylla pyricola*	Flower plant mixture, alleyways	Null	[144]
^2^ Pear	Psyllid	*Cacopsylla pyri*	Ash, ivy, polar hedgerow	Null	[147]
^2^ Apple	Spider mites	*Panonychus ulmi*	Flower plant mixture, alleyways	Null	[144]
^1^ Apple	Spider mites	*Panonychus ulmi*	Plant cover	Null	[153]
^2^ Vines	Lepidoptera	Tortricidae	Buckwheat	Null, Positive	[132]
^1^ Apple	General	Apple pests	Plant cover and or interplanted fruit trees	Null, Variable	[154]
^1^ Peach	General	Peach pests	Plant cover and or interplanted fruit trees	Null, Variable	[154]
^2^ Vines	General	Various	Flowers, alleyways	Variable	[137]
^2^ Apple	Various	Spider mites, Leucoptera malifoliella, codling moth, and Tortricidae	Flower plant mixture, alleyways	Positive, Null	[49]
^1^ Apple	Aphid	*Dysaphis plantaginea*	Flower strips	Positive	[155]
^1^ Apple	Aphid	*Aphis pomi, Dysaphis plantaginea*	Flower strips	Positive	[66,67]
^2^ Vines	Cicadellidae	Leafhoppers	Flower plant mixture, alleyways	Positive	[156]
^2^ Blueberry	General	Various	Flower plant mixture, margins	Positive	[95]
^2^ Apple	Homoptera	*Lygus*	Flower plant mixture, alleyways	Positive	[69]
^2^ Cherry	Homoptera	Aphid bait cards	Flower plant mixture, alleyways	Positive	[40]
^2^ Apple	Homoptera, Cicadellidae, Lepidoptera	*Dysaphis plantaginea*, leaf hopper, and codling moth	Flower plant mixture, alleyways	Positive	[29]
^2^ Apple	Homoptera, Formicidae	*Dysaphis plantaginea*, ants	Flower margins	Positive	[92]
^1^ Apple	Lepidoptera	Tortricidae	Peach nectaries	Positive	[157]
^2^ Strawberry	Lepidoptera	Acleris comariana, Tortricidae	Margin, buckwheat	Positive	[106]
^2^ Vines	Lepidoptera	Tortricidae	Margin, buckwheat	Positive	[105]
^2^ Apple	Lepidoptera, Hompotera	Codling moth, *Dysaphis plantaginea*	Flower plant mixture, alleyways	Positive	[50]
^2^ Apple	Lepidoptera, Hompotera	Codling moth, aphids	Flower plant mixture, alleyways	Positive	Fountain et al. (unpublished)
^1^ Pear	Psyllid	*Cacopsylla pyri*	Hedgerow	Positive	[158,159]
^1^ Pear	Psyllid	*Cacopsylla pyri*	Plant cover	Positive	[147]
^2^ Pear	Psyllid, Homoptera, Pseudococcidae	*Psylla chinensis, Aphis citricola*, and *Pseudococcus comstocki*	Aramatic plants, alleyways	Positive	[65]
^1^ Apple	Spider mites	*Tetranychus* spp.	Understory plants	Positive	[160]
^1^ Apple	Spider mites	Spider mites	Understory plants	Positive	[161]
^1^ Apple	Spider mites	*Panonychus ulmi*	Adjacent bushes	Positive	[162]
^1^ Apple	Spider mites	*Tetranychus* spp.	Plant cover	Positive	[163]
^2^ Apple	Heteroptera, Lepidoptera	*Lygus*, caterpillars	Flower plant mixture, alleyways	Positive	[47]
^2^ Apple	Homoptera	*Eriosoma lanigerum*	Flowers	Positive	[143]
^1^ Apple	Lepidoptera	Tortricidae	Buckwheat	Positive	[135]
^1^ Apple	Lepidoptera	Tortricidae	Alyssum	Positive	[135]
^1^ Apple	Lepidoptera	Tent caterpillar and codling moth	Understory plants	Positive	[131]
^1^ Apple	Lepidoptera	Tortricidae	Buckwheat, alleyways	Positive	[126]

**Table 3:** Effects of wildflowers or cover crop floral enhancements on insect pollinators and fruit production. The effect of plant manipulation on pollinator numbers and/or diversity is positive, null, or negative. NB: different effects may be due to species growth, location, or timing.

Fruit Crop	Target Pollinators	Plant Manipulation(S) or Presence	Location/Scale	Effect on Crop	Effect on Pollinator	Source
Blueberry (highbush)	Honeybees, wild bees, and hoverflies	15 perennial wildflower species	Margin	Fruit set, berry weight, mature seeds, yield greater in fields adjacent to wildflower plantings	Null (honeybees), positive (wild bees and hoverflies)	[164]
Apple (cider)	Honeybees, wild bees, and hoverflies	25 wildflower species	Alley	Increase visits to apple blossoms, fruit set	Positive (wild bees, Andrenid, and flies)	[121]
Mango	Pollinators	*Aloe greatheadii, Barleria obtusa*	Margin	Higher production	Positive	[52]
Strawberry	Honeybees, wild bees, and hoverflies	Annual and biennial fowering species	Margin	Not measured	Positive (wild bees and bumblebees)	[165]
Apple	Honeybees, wild bees, and hoverflies	Nine herbaceous species	Alley	None	Null (bees), positive (hoverflies)	[48]
Blueberry, sour-cherry, and watermelon	Wild bees	Enhanced floral margins	Margin	Not measured	Positive	[166]
Apple	*Osmia lignaria*	Bigleaf lupine, *Lupinus polyphyllus*	Margin	Not measured	Positive	[167]
Cherry (protected)	Pollinating insects	Perennial wildflower mix	Alley	Not measured	Positive	[94]
Cherry	Wild bees	Semi-natural habitat, including floral resources in orchards	Alley and landscape	Wild pollinator positive influence on fruit set	Positive	[53]
Apple	Honeybees	Semi-natural habitat including floral resources in orchards	Within orchard	Not measured	Positive	[43]
Cherry	Honeybees, wild bees	Non-intensively managed areas	Landscape	Increased bee resources from 20% to 50% enhanced fruit set by 150%	Positive (wild bees)	[54]
Apple	Wild bees (spring wild bees)	Local and landscape flora	Landscape and local	Not measured	Positive	[168]
Apple, cider	Wild pollinators	Landscape and small-scale orchard features	Landscape and local	Increased fruit set and seed set	Positive	[58]
Apple	Wild pollinators	Organic vs. integrated management	Margin, landscape	Reduced pollination deficit measured	Positive	[51]
Apple	Bumblebees	Hedgerows, flower strips	Landscape, margins	No consistent impact on fruit quality	Positive	[60]
Apple	Wild pollinators	Dandelion	Alley	Larger apples	Positive (apples), null (pollinators)	[169]
